# Food insecurity and nutritional vulnerability in women with cancer: financial toxicity as a driver of survivorship inequities

**DOI:** 10.3389/fpubh.2026.1905238

**Published:** 2026-07-28

**Authors:** Sihan Jing

**Affiliations:** School of Maritime Economics and Management, Dalian Maritime University, Dalian, China

**Keywords:** cancer nutrition, dietitian referral, financial toxicity, food insecurity, health economics, nutritional vulnerability, survivorship inequities, women with cancer

## Abstract

Food insecurity is an underrecognized determinant of nutritional vulnerability among women with cancer. Although oncology nutrition guidelines increasingly emphasize early screening, dietitian referral, body-composition preservation, and survivorship-oriented dietary patterns, many women experience financial toxicity after diagnosis through out-of-pocket medical costs, income loss, transportation expenses, caregiving disruption, and competing household needs. These economic pressures can restrict access to adequate, safe, and nutrient-dense foods, thereby worsening malnutrition risk, diet quality, sarcopenia, obesity-related metabolic dysfunction, fatigue, and treatment intolerance. The problem is especially relevant in breast, gynecologic, and other women's cancers, where endocrine therapy, menopausal transition, long-term survivorship care, and gendered caregiving roles may amplify nutritional and economic strain. This Mini Review synthesizes the health-economic pathway linking financial toxicity, food insecurity, and nutritional vulnerability in women with cancer. It discusses clinical mechanisms, evidence connecting food insecurity with care disruption and mortality, and implementation priorities for equitable nutrition care. A practical framework is proposed in which food insecurity screening, malnutrition assessment, financial navigation, dietitian referral, social support, and outcome monitoring are integrated into survivorship care. Equity should be evaluated not by service volume alone, but by whether women at greatest nutritional and financial risk receive timely, effective, and sustainable support.

## 1 Introduction

Nutrition is a modifiable determinant of cancer survivorship. In women with breast, cervical, ovarian, endometrial, and other cancers, nutritional status affects treatment tolerance, symptom burden, body composition, metabolic health, and quality of life (1–5). Yet nutrition is not only a matter of individual dietary choice. It is shaped by income, food prices, insurance coverage, employment security, caregiving demands, transportation, and the availability of oncology nutrition services. For this reason, food insecurity should be considered a central component of cancer nutrition research rather than a peripheral social variable.

Food insecurity refers to limited or uncertain access to nutritionally adequate and acceptable foods. In cancer care, it can emerge or intensify after diagnosis when patients face treatment-related costs, reduced work capacity, and repeated travel to oncology centers. Women may be particularly vulnerable because cancer treatment often intersects with household food management, unpaid caregiving, reproductive and menopausal health, and income interruption. Financial toxicity, broadly defined as the economic burden and distress caused by cancer care, is therefore a plausible upstream driver of nutritional vulnerability (6, 7).

This Mini Review focuses on the pathway through which financial toxicity contributes to food insecurity and nutritional vulnerability among women with cancer. It aims to align health economics with nutrition-oriented survivorship care by summarizing mechanisms, evidence, and implementation priorities. The central argument is that equitable cancer nutrition care requires simultaneous attention to food access, nutritional risk, and financial strain.

## 2 Financial toxicities as an upstream determinant of food insecurity

Cancer-related financial toxicity includes direct medical costs, non-medical costs, debt accumulation, employment disruption, loss of insurance, and psychological distress related to paying for care (8–10). Even in insured populations, copayments, deductibles, supportive medications, fertility preservation, transportation, parking, lodging, childcare, and nutritional supplements can generate substantial household strain. For women with cancer, the economic shock may be prolonged because survivorship often requires endocrine therapy, surveillance, management of late toxicities, rehabilitation, and chronic symptom control.

Financial toxicity can create food insecurity through several mechanisms. First, households may reallocate resources from food to medical care, transportation, or debt repayment. Second, treatment fatigue and income loss can reduce food purchasing and preparation capacity. Third, symptoms such as nausea, mucositis, diarrhea, taste change, pain, or early satiety may require specialized foods, oral nutrition supplements, or dietitian-guided meal planning, all of which may be unaffordable without coverage. Fourth, patients may avoid expensive nutrient-dense foods and rely on lower-cost, energy-dense, nutrient-poor foods. These trade-offs can produce the paradoxical coexistence of food insecurity, obesity, sarcopenia, and micronutrient insufficiency.

Food insecurity should therefore be interpreted as both an economic exposure and a nutritional exposure. It changes what patients can eat, when they eat, whether dietary advice is feasible, and whether symptom-responsive nutrition plans can be implemented. For women with cancer, financial toxicity may not simply worsen quality of life; it may alter dietary exposures that influence survivorship physiology.

## 3 Nutritional vulnerability in women with cancer

Nutritional vulnerability refers to the state in which dietary intake, body composition, metabolic reserve, or access to nutritional care is insufficient to support treatment and recovery. In oncology, this includes malnutrition, cachexia, sarcopenia, unintentional weight loss, obesity-related metabolic dysfunction, micronutrient inadequacy, and reduced dietary quality (3–5, 11–13). Women with cancer may experience these risks across multiple survivorship phases. During active treatment, symptoms may limit intake; after treatment, endocrine therapy, premature menopause, fatigue, and reduced physical activity may drive weight gain, adverse lipid profiles, insulin resistance, and body-composition change.

Food insecurity amplifies these risks by restricting access to fruits, vegetables, whole grains, lean proteins, dairy products, and culturally acceptable fresh foods (14–17). Reduced dietary diversity may weaken adherence to survivorship dietary recommendations and increase reliance on inexpensive ultra-processed foods. Such patterns can contribute to systemic inflammation, adiposity-related endocrine changes, impaired immune function, and metabolic dysregulation. In breast and endometrial cancer survivorship, these pathways are particularly relevant because obesity, insulin-IGF signaling, adipokines, and chronic inflammation have plausible links to recurrence risk, comorbidity burden, and quality of life (1, 2, 18–20).

Food insecurity can also disrupt clinical nutrition interventions. Dietitian advice to increase protein intake, consume small frequent meals, use oral nutrition supplements, or follow high-fiber dietary patterns may be unrealistic when food budgets are unstable. In this sense, nutritional counseling without food-access support may unintentionally widen inequities: women with adequate resources can implement recommendations, whereas those facing financial hardship may be labeled as non-adherent despite structural constraints.

## 4 Evidence linking food insecurity, care disruption, and survivorship outcomes

Evidence connecting food insecurity with cancer outcomes is growing. A scoping review found that food insecurity among cancer survivors is associated with financial hardship, poor diet quality, psychological distress, and unmet supportive care needs (21). In urban low-income cancer populations, food insecurity has been documented as common and clinically relevant (22). More recently, a US cohort study of cancer survivors reported that food insecurity was associated with higher all-cause mortality after full adjustment, with stronger associations in some income and assistance-program subgroups (23). Although causality cannot be inferred from observational data, these findings support routine screening and linkage to assistance as components of survivorship care.

Food insecurity is also associated with forgone medical care. Cancer survivors with persistent or recurrent food insecurity are more likely to delay, change, or forgo prescription medications and other care because of cost (24). This matters for women with cancer because oral endocrine therapies, antiemetics, pain medicines, bone-protective agents, and supportive treatments may be long-term and sensitive to cost-related non-adherence. Financial toxicity has independently been linked to lower quality of life, distress, bankruptcy, and mortality among cancer patients and survivors (7–10, 25–29). Together, these findings suggest a bidirectional cycle: cancer treatment creates financial strain; financial strain increases food insecurity; food insecurity worsens nutrition and adherence; clinical deterioration increases costs and deepens financial strain.

The equity dimension is central. Racial and ethnic minority groups, rural residents, women with lower income, single parents, immigrants, older adults living alone, and patients without stable insurance may be exposed to multiple disadvantages. Food insecurity can therefore mediate the relationship between structural inequity and survivorship outcomes. For oncology nutrition, the goal should not be universal delivery of identical advice, but proportionate support based on financial, nutritional, and social risk.

## 5 Screening and integrated care pathways

A practical response begins with measurement. Food insecurity can be screened with brief validated tools, including the two-item Hunger Vital Sign, while nutritional risk can be assessed using instruments such as the Malnutrition Screening Tool, Patient-Generated Subjective Global Assessment, Nutrition Risk Screening 2002, or GLIM-based criteria (4, 5, 30, 31). Financial toxicity may be assessed using patient-reported measures such as the Comprehensive Score for Financial Toxicity or through structured questions about cost-related distress, debt, employment disruption, and medication trade-offs (9, 32).

Screening should not occur in isolation. Positive screens require a response pathway that includes oncology dietitians, social workers, financial navigators, patient navigators, and community food resources. Women with high nutritional risk and food insecurity may need rapid dietitian referral, symptom-responsive meal planning, oral nutrition support when clinically indicated, grocery vouchers, medically tailored meals, food pantry linkage, benefit enrollment, and transportation support (33–36). Patients with lower risk may benefit from budget-sensitive dietary education, culturally appropriate food lists, and digital follow-up.

Service allocation should be equity-oriented. Efficiency should not be measured only by the number of nutrition consultations delivered. A more meaningful metric is whether women at highest risk receive timely and effective care. Relevant indicators include screening completion, time from positive screen to referral, dietitian access, food assistance uptake, weight stabilization, dietary quality, treatment completion, patient-reported quality of life, and financial distress reduction. Key health-economic drivers of nutritional vulnerability and corresponding equity-oriented responses are summarized in Table 1.

**Table 1:** Health-economic drivers of nutritional vulnerability and equity-oriented responses.

Driver	Nutrition-related pathway	Women's cancer relevance	Equity-oriented response
Out-of-pocket medical costs	Resources shift from food to care, medications, travel, or debt.	May reduce ability to purchase protein-rich foods, oral supplements, and fresh foods during treatment.	Screen for financial distress; offer financial navigation and benefit enrollment.
Employment and income disruption	Reduced household income limits food quantity and dietary diversity.	Particularly relevant for working-age women and single-parent households.	Coordinate work-support resources, social work referral, and food assistance.
Treatment-related symptoms	Specialized foods or symptom-responsive meal plans may be unaffordable.	Nausea, mucositis, fatigue, diarrhea, and taste changes can impair intake.	Rapid dietitian referral with low-cost symptom-specific meal strategies.
Food insecurity	Meal skipping, lower fruit/vegetable intake, low protein intake, and reliance on ultra-processed foods.	May worsen malnutrition, sarcopenia, obesity, fatigue, and treatment tolerance.	Use brief food-security screening and connect to vouchers, pantries, or medically tailored meals.
Fragmented service delivery	Nutrition, financial, and social needs are addressed separately or not at all.	High-risk women may receive advice without feasible implementation support.	Integrate nutrition screening, social support, and outcome monitoring in survivorship care.

## 6 Health-economic and policy priorities

The health-economic case for addressing food insecurity in cancer survivorship rests on value, not only compassion. Nutritional vulnerability can increase hospitalizations, treatment interruptions, emergency care, and supportive medication use. Conversely, targeted food and nutrition interventions may improve treatment completion, quality of life, and care efficiency. A randomized trial of food insecurity interventions in safety-net cancer clinics found that food support was associated with improvements in food security, depression, quality of life, and treatment completion (33). Although evidence remains incomplete, these data suggest that food assistance can function as an oncology supportive-care intervention.

Future research should evaluate cost-effectiveness and implementation across diverse settings. Priority questions include which patients benefit most from food support, whether interventions should emphasize vouchers, pantry access, medically tailored meals, cash transfers, or dietitian-led nutrition prescriptions, and how programs should be adapted for women undergoing chemotherapy, endocrine therapy, radiotherapy, surgery, or palliative care. Studies should report not only food security status but also dietary intake, nutrition biomarkers, body composition, treatment adherence, patient-reported outcomes, and survival.

Policy priorities include insurance coverage for medical nutrition therapy, reimbursement for oncology dietitian services, integration of food insecurity screening into cancer quality metrics, and partnerships between cancer centers, public health agencies, food banks, and community organizations. For low- and middle-income countries, the same framework should be adapted to local food systems, informal caregiving structures, and catastrophic health expenditure patterns. Ultimately, financial toxicity and food insecurity should be treated as modifiable barriers to effective cancer nutrition rather than as background socioeconomic descriptors.

## 7 Discussion and conclusion

**Figure 1:**
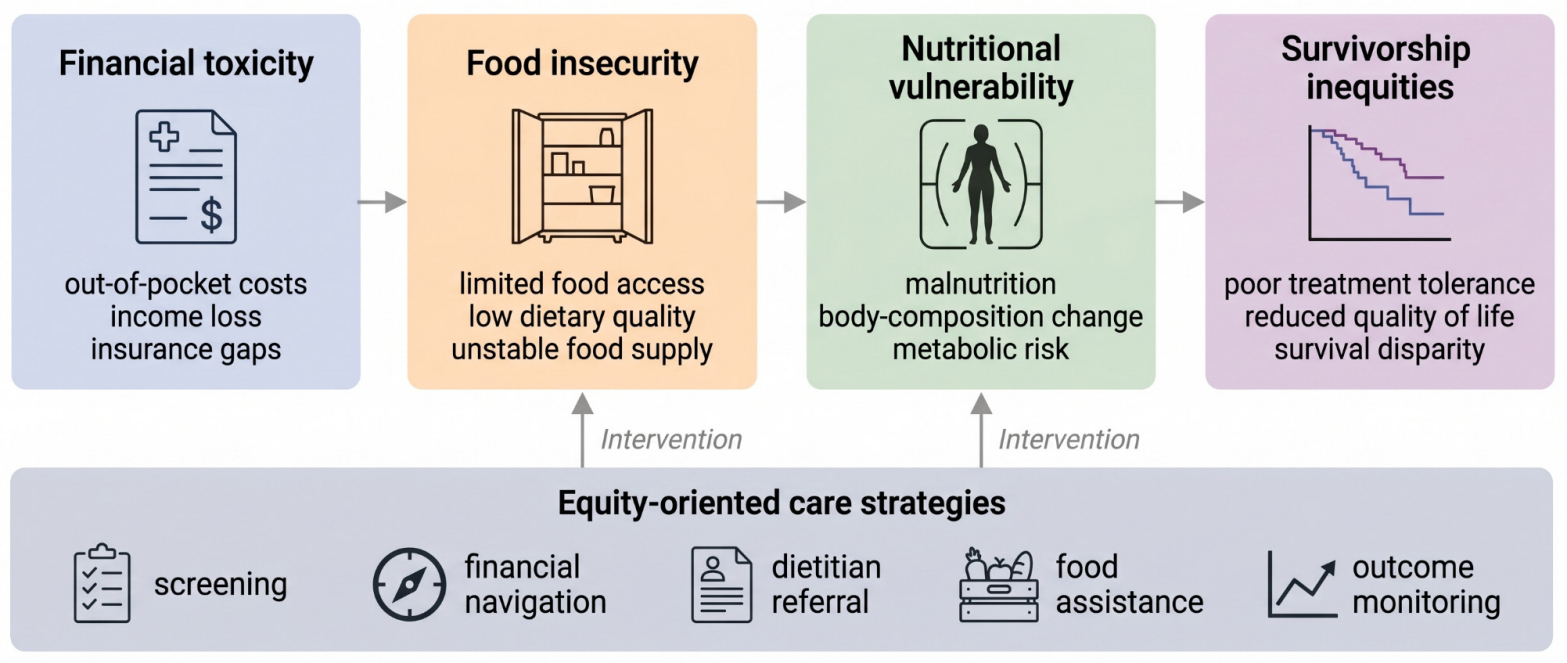
Health-economic pathway linking financial toxicity, food insecurity, and nutritional vulnerability in women with cancer. Financial toxicity can restrict food access and undermine dietary quality, increasing nutritional vulnerability through malnutrition, poor body-composition outcomes, and metabolic risk. Equity-oriented care requires screening, financial navigation, dietitian referral, food assistance, and outcome monitoring.

Food insecurity and nutritional vulnerability provide a bridge between health economics and cancer nutrition. In women with cancer, financial toxicity can shape dietary exposures, adherence to nutrition advice, symptom management, and long-term survivorship health. The pathway is clinically plausible and increasingly supported by population-level evidence, but it remains under-integrated into routine oncology care. A conceptual synthesis of this pathway is illustrated in Figure 1.

This Mini Review proposes a shift from advice-centered nutrition counseling to equity-oriented nutrition care. Such care should identify food insecurity, measure nutritional risk, evaluate financial toxicity, and connect women to timely dietetic and social support. The implication for research is equally clear: studies of nutrition and women's cancer survivorship should incorporate economic and food-access variables, while studies of financial toxicity should measure dietary and nutritional consequences.

The practical conclusion is that food insecurity is not merely a social determinant adjacent to cancer nutrition; it is a direct determinant of whether nutrition recommendations can be implemented. Reducing survivorship inequities among women with cancer will require nutrition care pathways that are clinically precise, financially realistic, and socially accountable.
